# The experiences of Red Crescent relief workers during the COVID-19 pandemic in Iran: a qualitative phenomenological study

**DOI:** 10.1186/s12913-023-09920-8

**Published:** 2023-09-06

**Authors:** Minoo Mohammadkhani, Mohammad Sadegh Tavakoli Sani, Arezoo Sarani, Narges Khanjani

**Affiliations:** 1 Student Research Committee, Kerman University of Medical Sciences, Kerman, Iran; 2 Health in Disasters and Emergencies Research Center, Institute for Futures Studies in Health, Kerman University of Medical Sciences, Kerman, Iran; 3 Department of Medical Education, Paul L. Foster School of Medicine, Texas Tech University Health Sciences Center El Paso, El Paso, TX 79905 USA

**Keywords:** Red Crescent Society, COVID-19, Qualitative research, Phenomenology

## Abstract

**Background:**

The daily increase of infected individuals and mortalities related to COVID-19 in Iran increased public fear and anxiety and affected the job performance of many health workers, including the Iranian Red Crescent Society, as one of the organizations responding to COVID-19.

**Methods:**

This study aimed to explore the experiences of Red Crescent rescuers, using a qualitative method with an interpretative phenomenological analysis (IPA), and by conducting semi-structured and in-depth interviews with Red Crescent relief workers from Mashhad in September 2020. Participants were selected by purposive sampling.

**Results:**

Rescuers in the Red Crescent Society, had gained different experiences during the COVID-19 pandemic. The experiences were categorized into four main themes. These main themes were: (1) Psychological disorders, (2) Organizational support (3) Mis-management (both structural and human factors), and (4) Opportunities.

**Conclusion:**

The COVID-19 epidemic did create unique opportunities to understand the pitfalls of the Red Crescent aid services. Red Crescent rescue workers suffered from psychological symptoms, and mismanagement was also present. Psychological support and organizational improvements should be implemented.

## Background

Coronavirus disease 19 (COVID-19), originated in Wuhan Province in China in December 2019 [1–3], and became a major health crisis. On January 30, 2020 [4], the World Health Organization declared it a public health emergency of international concern [5].

Although most of the symptoms of COVID-19, such as fever, cough, and fatigue, are similar to those of other coronavirus diseases such as SARS and MERS, many people can be infected with the virus yet asymptomatic, and this causes high unintentional and hidden disease transmission [6]. As a result, governments around the world enacted unprecedented public policies such as social distancing, segregation, and quarantine to prevent the spread of disease [7].

Given the nature of this pandemic, the contradictory news, and the policies adopted to prevent morbidity and mortality, the disease has had far-reaching and unprecedented economic and psychosocial consequences worldwide [1, 7].

Following the outbreak of diseases such as SARS, influenza and COVID-19, adverse mental health consequences such as fear, anxiety and post-traumatic stress disorder have been observed, especially among the patients [8]. Fear is defined as an unpleasant emotional state caused by perceiving threatening stimuli [9]. Understanding the sensitivity of the issue can lead to an understanding of danger, preventative measures and feelings such as fear and anxiety, which can lead to unusual behaviors. The association between fear and health-related behaviors is complex, as fear has been used to change people’s attitudes and behaviors on various subjects (e.g., smoking, breast exams, sunscreen use, and regular medication use) [7].

Fear of infection causes individuals to avoid behaviors that may put them at risk for the disease, and above all, fear of COVID-19 is a breeding ground for social stigma that has led individuals to hide their illness [1, 7]. These conditions can have psychological effects on people worldwide. For instance, a recent study in Canada reported that from 1354 participants, one-third of them were concerned about COVID-19 [7]. In addition, in an online survey of 808 adults in the USA, 56% of participants reported they were concerned or very concerned about the prevalence of COVID-19 [7]. Another study in the US reported that individuals were more concerned about COVID-19 than seasonal flu (37% vs. 27%) [10]. McGlinchey et al. reported that the mental state of health care professionals was affected during the COVID-19 pandemic, and many of them had experienced feelings of fear and sadness in addition to physical fatigue [11].

The Red Crescent Society, as a non-governmental humanitarian organization, consists of volunteers and employees who provided various services and also participated in the fight against COVID-19, by screening the population to identify infected patients [12]. However, by the time this study was conducted no research was done on Red Crescent relief workers, with a qualitative approach, to examine their experiences, the challenges ahead and the support strategies of the organization. Documenting their experiences can help find appropriate solutions to improve the performance of Red Crescent rescuers in the future.

## Methods

### Study design

A qualitative interpretative phenomenological analysis (IPA) was used to explore the experiences of Red Crescent rescuers during COVID-19 [13, 14]. Although descriptive and interpretive phenomenology are both systematic thematic-descriptive methods, they follow different approaches. In descriptive phenomenology, researchers focus on the interdependent components of the phenomenon’s structure, and it is done in situations where there is little or ambiguous information about the phenomenon, or we want to explore the different dimensions of a phenomenon. But in an IPA, thematic analysis is done and by recording the specific experiences of specific people, authors look for the meaning and interpretation of the phenomenon in a specific context [15, 16].

### Sampling and study setting

A total of seven Red Crescent rescuers (5 employees and 2 volunteers) were chosen and enrolled by purposive sampling from Mashhad, a city in northeast Iran, in September 2020 (during one month). Interviews were continued until data saturation. We received no new information after the fifth interview and saturation was achieved, but to be sure we also did two additional interviews. Each participant was interviewed once.

After explaining the purpose of the study, informed verbal consent was obtained from all participants, including permission to record the conversation. In order to remain anonymous, a study ID was assigned to each participant at the time of enrollment. The interviews were conducted using a semi-structured questionnaire. Interviews were conducted at a place that was more convenient for the participants. Some were interviewed at the relief base and some at the population support center. Interviews were done face to face. This study was approved by the Ethics Committee of Kerman University of Medical Sciences [Ethics code: IR.KMU.REC.1399.310].

### Recruitment

The interview questions were framed based on the aims of the study, the viewpoints of the research team and pilot interviews with two officers from the Red Crescent population.

Participants were selected from those volunteers who had the most attendance and work shifts at the beginning of the epidemic. Relief workers who did not want to participate were excluded.

### Interview

Interviews were conducted using the funnel approach. In this technique, the interviewer starts by asking more general questions, and then moves on to more and more detailed and specific questions [13]. The interview questions were designed to be flexible, neutral and without direction. The outline of the interview questions was based on the purpose of the study, the opinion of the research group, and also the pilot interviews. The interviewer (second author) was a PhD student majoring in disaster health. He completed the necessary courses for qualitative research, and the interviews were conducted under supervision of the fourth author. Because the interviewer had previously worked in this organization, he was able to communicate well with the rescuers. Before starting the questions, the reasons for conducting the research and the importance of knowing the experiences of the rescuers during the epidemic period were explained to the participants. Participants consented to their voices being recorded. Each interview lasted 20 to 45 minutes. Field notes were made during the interviews, and participants were encouraged to explain further by asking appropriate questions after the initial questions. Moreover, new questions were developed based on previous questions. The main questions asked were as follows: (1) How did you feel during the COVID-19 pandemic? (2) What changed in your performance in that situation? (3) What support should have the organization provided?

### Data analysis and rigor

The purpose of this study was to explore and interpret the lived experiences of Red Crescent rescuers during the COVID-19 pandemic, and therefore IPA was used for analysis [17].

In this study, the 6-steps process described by Smith et al. was used, as follows: 1- The interviews were transcribed verbatim to understand the rescuers’ experiences fully, and the texts were read several times, 2- Key quotations were identified, 3- A list of codes was extracted according to the key quotes and impressions recorded from each transcript, 4- The relations between the codes were identified, 5- This process was performed for each transcript. 6- In order to form the final themes, similarities and differences were identified among all codes [13, 14].

According to Lincoln and Guba (1985), four criteria of credibility, transferability, dependability and confirmability were used to ensure validity and reliability.

With the presence of the researcher in the research environment and spending enough time, the dependability of the results was obtained. Authors increased the transferability of data through in-depth description and providing direct quotes, choosing the right people to interview, collecting and simultaneously analyzing data. Credibility was achieved by returning the transcripts to the participants. The concepts extracted from each interview were presented to the interviewees and their opinion was asked regarding the correct understanding of the concepts. Finally, to ensure the confirmability of the results, transcripts and the created themes and sub-themes were reviewed and checked by a few qualitative researchers, and their suggestions and comments were applied [18].

## Results

All participants were male, their mean age was 37.1 years and their work experience was 11.0 years on average. The demographic characteristics of the participants have been shown in Table 1.

**Table 1:** The demographic characteristics of the participants

Code of participant	Mean age	Mean work experience
Participant number 1	44 years	17 years
Participant number 2	46 years	10 years
Participant number 3	37 years	18 years
Participant number 4	40 years	8 years
Participant number 5	35 years	15 years
Participant number 6	30 years	5 years
Participant number 7	28 years	4 years

The experiences of Red Crescent rescuers during the COVID-19 pandemic were categorized into four main themes. These main themes were: (1) Psychological disorders, (2) Organizational support (3) Mismanagement (both structural and human factors), and (4) Opportunities. The themes and sub-themes have been shown in Table 2.

**Table 2:** Themes and sub-themes extracted from the interpretative phenomenological analysis

Theme	Sub theme	Code
Psychological disorders	Fear	Morbidity, transmission
	Stress	Financial, occupational
	Obsession	Obsessive-Compulsive Disorder
Organizational support	Intangible Supportive services	Consulting services, Education
	Logistics services	Provision of influenza vaccine, Provision of personal protective equipment
Mismanagement	Structural factors	Unfair distribution of human and material resources
	Personnel factors	Feeling discriminated against emergency services workers
Opportunities	Reduction of rescue operations	Decrease in road accidents
	Learning full compliance with health protocols	Social distancing Wearing a mask
	Revealing system weaknesses	The need for better supervision and training of rescuers

### Psychological disorders

The COVID-19 pandemic seemed to have a negative effect on the mental health and emotions of people, especially high-risk occupations such as physicians, nurses, and so on.

*“Life has become such a meaningless thing, it is bothering, and this is occupying a large part of our mind”* (P; 3).

Our study showed that COVID-19 resulted in fear and stress among rescuers so that the enthusiasm for the job before the pandemic had turned into compulsion and a sense of responsibility. Fear of being infected made it difficult for rescuers to go to the workplace, and affected their lives. Some stated that the only reason they went to work was financial issues. Most participants pointed out that they have better coped with their fear and anxiety over time, and as they gained more information about the disease.

*“We were very eager to go to the workplace before…but now …, because we think some percentage of the injured are infected … we go because we have to…”* (P; 5).

Moreover, due to the infection of colleagues, the increase of carriers and non-compliance with protocols in the workplace, rescuers were worried about becoming ill with COVID-19 and unknowingly transmitting the disease to family members. Due to some predisposing factors for the disease, volunteers with underlying diseases or retirees who served as volunteers in the population were unwilling to cooperate after the epidemic.

*“I feel some changes have happened, that is, those who were in the workplace, now leave work due to fear of COVID-19. Some retired colleagues resigned …”* (P; 2).

From the participants comments it seemed like, due to the fear of being infected, the accuracy and sensitivity of health workers in caring for injured patients, especially those who had respiratory symptoms, had decreased.

On one hand, participants were obsessed with infection in their environment, and on the other hand, they constantly considered decontaminating the environment as a time-consuming and costly task, and this dual feeling challenged them.

*“I always think the environment is infected and because of this I don’t work as always…. I think previous shift employees have not observed the protocols, and the equipment is infected. Therefore, if I want to touch something, I have to clean everything thoroughly, disinfect it, and then touch it”* (P; 5).

### Organizational support

Organizational support was needed in both intangible issues such as education and psychological support, and logistics such as providing the vaccine and protective equipment.

The participants commented that because job stress has doubled with the onset of the epidemic, organizations should pay more attention to raising awareness about the disease, how to prevent it and the psychological problems of rescuers; and they should provide them with consulting services.

*“Exposure to the injured, and the dead [from COVID-19], has adverse psychological effects on the volunteers”* (P; 4).

Providing the influenza vaccine (because of its possible effect in preventing different types of influenzas and COVID-19) and personal protective equipment (PPE) were the most important management strategies suggested to reduce stress among red crescent rescuers.

*“Because we are in contact with the injured [and they might be infected], one of our needs is to receive the influenza vaccine. It is necessary under the current conditions”* (P; 4).

### Mismanagement

Red crescent rescuers felt discriminated in receiving PPE compared to other health workers (e.g., emergency department (ED) workers). They believed that PPE, including coveralls and face shields, were available for ED workers sufficiently, but red crescent rescuers had to use the same equipment several times. They also said that face shields were not distributed among rescuers or were distributed in limited numbers after the section manager did a follow-up. But these shields were given sufficiently to volunteers who walked door to door and aimed to prevent the disease by inviting people to follow protocols.

Participants cited the lack of janitors in the Red Crescent as another difficulty of their work. They were also concerned about shifting workers, as each rescuer was in contact with a different person in each shift, compared to ED workers who worked in permanent places. Hence, the feeling of fear and risk of infection was higher among rescuers.

*“Rescuers in the Red Crescent relief base are not permanent, while workers in ED are permanent, for example, one of the EDs has six workers, and this number is stable each month, while in each shift we have to work with different individuals/rescuers”* (P; 3).

*“Recently, shields were given to some volunteers but not to us”* (P; 3).

### Opportunities

One of the positive consequences of the COVID-19 epidemic, which two participants noticed, was a reduction in rescue operations such as road accidents, which was because of the quarantine and less travel during this period.

*“We haven’t done many rescue operations since COVID-19 because the number of accidents and rescue trips has decreased”* (P; 1).

*“The number of dispatches of the workforce has decreased… maybe by even half”* (P; 3).

The COVID-19 pandemic caused rescuers and patients who previously did not observe health protocols to do so seriously. Participants pointed out that before the COVID-19 epidemic, there were protocols and guidelines for health care workers in dealing with the injured that many people may not have taken seriously. But after the epidemic and perceiving the risk of the disease, wearing masks was observed by the patients. Those who did not wear a face mask were required to do so when they came to the workplace.

*“In the past, when we went to the workplace, they asked us to wear a mask because maybe 1% of those we were in contact with were infected with the virus, but we didn’t wear a mask. Now we do that.”* (P; 5).

Another positive effect of the pandemic was that it helped reveal many of the weaknesses of the health system. The rescuers believed that there was poor supervision over the shifts. It seems that the authorities should pay more attention to supervision and training, especially in shifts. During the interview, one of the participants stated that there was no supervision over the staff and rescuers, and some rescuers came to work, despite being infected with COVID-19.

*“One of the employees infected with Corona was not willing to be absent from work due to the fact that the organization did not change his shift and on the other hand because of the loss of overtime payments related to his shift”* (P; 2).

A participant claimed that she was not aware of the existence of instructions for prohibiting sick people from coming to work, or their implementation and follow-up, which indicates the need for more training from the organization.

*“Their leave has not been defined, and we did not have any instructions for implementation. Either we did not have, or they did not implement, or they didn’t do a follow-up  [who knows which one it is?]”* (P; 2).

*“The protocol is followed at the base, but not at the posts. Still, there is no protocol for the posts, and they have not been given instructions about taking leave, if one was sick”* (P; 2).

A participant also stated that there was no supervision at the base (headquarters), and instructions were not followed.

*“Distance [between people] is not observed at the base, and almost no one wears masks”* (P; 1).

## Discussion

### Psychological disorders

COVID-19 has had far-reaching and unprecedented economic and psychosocial consequences worldwide [1, 7]. Iran is one of the countries that have been severely affected by COVID-19. As a non-profit and philanthropic organization, the Red Crescent Society is one of the organizations involved in disease and trauma control. Several studies have examined the effects of COVID-19 [1, 19–21], especially on the mental health of health care workers, but at the time this article was written, only one study from Qatar, with a different approach than ours (a cross-sectional study utilizing a web-based survey), had examined the psychological impact of the COVID-19 epidemic on the health of the workers of the Red Crescent Society [20]. Hence, the experiences of rescuers during the COVID-19 pandemic can help improve the services of this organization.

Epidemics have always had an impact on people’s mental health. The epidemic of an emerging disease can cause excessive and unusual stress due to concerns for the health and safety of oneself and family members, especially for health employees who are more exposed to infected people because of their jobs. This fear and stress can seriously affect psychological health [22]. Koh et al. (2005) reported that a high percentage of health care workers experienced an increase in workload and higher levels of anxiety during the SARS epidemic [23]. Mental health seems to have been affected during the current epidemic because of the restrictions and maintaining social distancing [24] as well.

Studies such as Petrie et al. among Australian paramedics, Xu et al. on front-line nurses in Wuhan, China, Chen et al. on Chinese medical staff, and Maiorano et al. on medical staff (physicians and nurses) and emergency workers (firefighters, civil protection workers, and ambulance personnel) in Italy, have shown the significant adverse effects of COVID-19 on the mental health of health workers. During the pandemic, most paramedics in Australia reported symptoms of burnout and anxiety, frontline nurses in Wuhan reported stress and worry during rescue, and Chinese medical staff reported symptoms of psychological distress [25–28]. Maiorano et al. showed that emergency workers, and especially health care workers who worked with COVID-19 patients, were exposed to sources of high stress and risk of secondary trauma; and nurses and doctors had higher levels of stress compared to the emergency workers [28].

Researchers think there are risk factors and/or protective factors that can determine the level of psychological adaptation in epidemic conditions. In the study of Fonseca et al., 66% of emergency medical service personnel had good psychological adaptation to epidemic conditions, and in another study on Portuguese medical paramedics, the overall fear of COVID-19, had an average of 1.92 ± 0.94 from 4 and was interpreted as low and medium level fear. Psychological adaptation was related to their experience and overall mental health, as well as security and support measures [29–30]. Direct contact with COVID-19 patients, lack of PPE, and unforeseen events, were among the factors leading to stress in healthcare workers in another study [28].

Our results showed that the emerging virus and the unpredictable risk of disease, the uncertainty of the patients’ status in terms of infection, especially those with respiratory symptoms, infection of co-workers with the disease, non-compliance with protocols at bases and shifting the workers every month, and as a result, the contact of different rescuers with each other, the possibility of transmitting the disease to the family members, and the presence of predisposing factors in some volunteers, led to fear and job stress among rescuers. In our study, rescuers had to work despite their fear and stress, because of their sense of responsibility and/or financial issues. A study in China reported that despite the concern of health care workers about transmitting the disease to their family members, a sense of responsibility and sacrifice motivated them to come to work [19]. In contrast, a study by Balkhi et al. in Pakistan showed that during the COVID-19 pandemic, the participants had pretended to be sick to avoid going to their workplace or considered quitting or applying for a leave and had limited their social relations with people to ensure their safety [31]. In our study, unlike the study of Balkhi et al., some rescuers went to work to receive their surplus salary, despite having symptoms or being infected with COVID-19; and this also caused fear and stress for other employees.

A study in Qatar showed that the prevalence rates of anxiety and stress symptoms among Red Crescent healthcare workers were 14.2 and 18.5%, respectively, which was lower than reported in other studies. Researchers thought this was because of the relief activities in the organization and the fact that these rescuers had more resilience than other people in the community [20]. In contrast, our findings showed that rescuers feared that they might transmit the disease to their family members, due to the very contagious nature of the virus and its transmission from asymptomatic carriers. Furthermore, in a study conducted in Qatar, high-risk perception was an independent risk factor for negative mental health outcomes [20], which is in line with our results which found high-risk perception can lead to fear and stress among rescuers.

In our study, obsessive-compulsive behavior also appeared among rescuers, which has been shown by other studies to have increased among people with a history of this disorder, and in the general population during the early stages of the pandemic, as well [32]. In Ahmed et al.’s study during COVID-19, the HCWs in Egypt, reported higher moderate and severe OCD than the general population. In China, the medical staff showed more obsessive-compulsive psychological symptoms (25.6%). In another study from China, medical and healthcare workers (5.3%) had a higher prevalence of obsessive-compulsive symptoms than nonmedical/health workers (2.2%) [33–35].

### Organizational support and mismanagement

Training is a key element in coping with emerging epidemics, as fear decreases by increasing knowledge about infection prevention and control methods [19]. In addition, having clear instructions and guidelines makes people feel safe [20]. In our study, participants pointed to education and monitoring as an important duty of the organization. Vagni et al. also showed that training programs and supervisory interventions are important protective factors for preventing secondary traumas among emergency workers [36]. A study by Tan et al. in Singapore showed that non–frontline nurses and the general public had more vicarious traumatization than frontline nurses, and the reason for this was the lack of sufficient psychological support from the relevant organization and less intensive training on PPE and infection control measures [21].

Chen et al. took care of the psychological pressures of medical workers by conducting a psychological intervention, through online or telephone group activities. In the same study, some medical staff were concerned about the lack of protective equipment, and the hospital created strict rules about the use and management of protective equipment to reduce these concerns, and also for more support, the hospital authorities had provided a place to temporarily separate infected employees from their families, and provided their daily needs as well [27]. But in Petrie et al.’s study, the paramedics in Australia were dissatisfied with supervisor and co-worker support, adequacy of training, and the frequency of protocol changes. Also, inadequate PPE caused distress among health care workers [25].

Using PPE is highly important during the pandemic, and the media has also emphasized this issue [37]. In our study, participants felt discrimination regarding PPE distribution compared to others, especially those working in the ED. They also believed that wearing face masks, as the most effective protective factor against the virus, is boring in the long term, causing staff to not use it. Some believe that the long-term use of masks can lead to dizziness due to decrease in blood oxygen levels, and frequent rebreathing of carbon dioxide [38]. Furthermore, given the change in working shifts, the rescuers were in contact with different individuals, which increased their concern.

This study showed that relief workers in critical situations need to learn strategies to control their stress, and need fair management, without discrimination, to address their needs.

The role of organizational managers and supervisors in supporting employees, especially those who are under more stress, as well as understanding and responding to their health, safety and occupational well-being concerns, is important. Managers should be trained on how to support their employees [22].

Xu et al. stated that managers should conduct serious training of infectious disease control for medical staff, especially emergency response teams, to generate the necessary preparedness for emergency situations, as Petrie et al. also pointed out the important role of workplace support as a protective factor for mental health and reducing stress (25–26). A qualitative study in China showed that the organization can play a vital role in reducing stress among healthcare workers by providing a safe work environment, adequate protective equipment and having personnel responsible for training, continuous monitoring and supervision, as well as supportive conversations and recommendations [19].

Therefore, the organizations should provide adequate protective equipment and create a safe and healthy environment to reduce stress and fear among staff. Employers are required to respond to COVID-19 within the framework of occupational health and safety legislations. Occupational Health and Safety Act No. 85 of 1993 obliges employers to provide and maintain a safe working environment [39]. Certainly, the support of the organization can increase the quality of the rescuers’ work.

### Opportunities

In our study, participants also pointed to the opportunities created by the COVID-19 pandemic, one of which was the reduction of accidents, due to restrictions and quarantine. The number of deaths caused by road accidents reported by the Iranian Forensic Medicine Organization in the Nowruz holidays (Persian New Year) of 2019 was 914 deaths, but after the start of the pandemic in 2020 in the same time of the year (Nowruz holidays) the number of deaths decreased to 534 [40]. Some other world studies also stated that road traffic accidents decreased during the COVID-19 restrictions, which was because of the decreased number of cars on the roads. This was observed in European countries, especially in France, Spain and Italy, where the greatest reduction in road traffic collisions occurred in April 2020 compared to April 2019. The restrictions during the pandemic also caused a significant reduction in traffic accidents in the province of Tarragona in Spain [41, 42].

Another item extracted from our interviews, was better compliance with instructions and protocols that were previously mentioned only theoretically, and not strictly followed in practice. From the beginning of the pandemic, more efforts had been made to fully adhere to the protocols. It seems like fear and the unknown nature of the disease at the beginning of the pandemic forced people to follow the protocols. It was emphasized in a study on bank employees that awareness and attitude affect the implementation of preventive measures and their effectiveness [43], and as stated by Lotfollahzadeh et al., the commitment to implement the compiled protocols is required to be monitored by managers [44].

By interpreting the experiences of paramedics in this study, it can be concluded that with the commencement of critical situations such as pandemics, the need for psychological support and proper management and supervision is felt more and more, especially among health care workers and paramedics. Therefore, the plans and policies of health care organizations need to be adjusted in such a way that they prioritize these issues.

One of the strengths of this study was that it was done during the pandemic and people who were directly involved with the crisis, were interviewed. Also, the method of conducting this study allowed the participants to speak about their experience and provide information without limitation. This study included different participants with at least four years of work experience, so the interviewees were able to compare their work experience during the epidemic with the years of work in the past, and as a result, they understood the problems better.

The results of this study show the pitfalls that need to be taken care of in the Red Crescent Organization and can help better manage the next pandemics.

## Conclusion

The COVID-19 epidemic did create unique learning opportunities to understand the experiences of rescuers and the challenges they face in crises. Red Crescent rescue workers, like other health care workers, suffered from psychological symptoms during the COVID-19 pandemic, and mismanagement made the situation worse. Therefore, in order to prevent reduction in the quality of rescuers’ work, psychological support, proper organizational management, and supervision are required.

## Data Availability

The datasets analyzed during the current study are available from the corresponding author on reasonable request.

## Abbreviations

IPA: Interpretative Phenomenological Analysis
COVID-19: Coronavirus Disease 19
PPE: Personal Protective Equipment
ED: Emergency Department
